# Female-female aggression and male responses to the two colour morphs of female common cuckoos

**DOI:** 10.1007/s00114-020-01680-3

**Published:** 2020-06-20

**Authors:** Csaba Moskát, Márk E. Hauber, Jana Růžičková, Attila Marton, Miklós Bán, Zoltán Elek

**Affiliations:** 1 MTA-ELTE-MTM Ecology Research Group, a joint research group of the Hungarian Academy of Sciences, the Biological Institute of the Eötvös Loránd University and the Hungarian Natural History Museum, MTM, Baross u. 13., Budapest, H-1088 Hungary; 2 Zoological Department, Hungarian Natural History Museum, Baross u. 13., Budapest, H-1088 Hungary; 3 Department of Evolution, Ecology, and Behavior, School of Integrative Biology, University of Illinois at Urbana-Champaign, 505 S. Goodwin Avenue, Urbana, IL 61801 USA; 4 Department of Evolutionary Zoology and Human Biology, University of Debrecen, Egyetem tér 1., Debrecen, H-4032 Hungary; 5 Juhász-Nagy Pál Doctoral School, University of Debrecen, Debrecen, Hungary; 6 MTA-DE Behavioural Ecology Research Group, Department of Evolutionary Zoology and Human Biology, University of Debrecen, Egyetem tér 1., Debrecen, H-4032 Hungary

**Keywords:** Acoustic playback, Colour polymorphism, 3D model, Female-female aggression, Territory

## Abstract

Female-only colour polymorphism is rare in birds, but occurs in brood parasitic cuckoos (Cuculidae). Obligate brood parasites leave incubation and parental care to other species (hosts), so female-female interactions can play a role in how parasites guard critical resources (host nests) within their laying areas. The plumage of adult female common cuckoos (*Cuculus canorus*) is either rufous (typically rare) or grey (common), whereas adult male conspecifics are monochromatic (grey). In previous studies, hosts and conspecific males responded with less intensity toward the rare female morph in support of a negative frequency-dependent benefit of female plumage polychromatism. Here, we assessed responses of both conspecific females and males to vocal playbacks of female calls, coupled with one of two 3D models of the different morphs of female cuckoos. At our study population, the rufous female morph was as common as the grey morph; therefore, we predicted similarly high rates of conspecific responses in both treatments. Both female and male cuckoos responded to playbacks acoustically, which demonstrated the primary role of acoustic communication in social interactions amongst cuckoos. Following this, some cuckoos flew closer to the models to inspect them visually. As predicted, no significant differences were detected between the live cuckoos’ responses toward the two colour morphs in this population. We conclude that dichromatism in female cuckoos evolved to serve one or more functions other than conspecific signalling.

## Introduction

Colour polymorphism (or polychromatism) refers to the existence of two or more discrete spectral phenotypes of individuals in a population (Caro 2005; Roulin 2004; White and Kemp 2016). Such polymorphism may be controlled genetically and/or developmentally and may vary between populations, habitats, sexes, life-history stages, and/or age classes. Animal colour polymorphism occurs in diverse invertebrate (Ajuira-Ibarra and Reader 2013) and vertebrate (Hubbard et al. 2010) lineages. Colour morphs may be adaptive for concealment, interspecific and intraspecific communication (which includes sexual selection), and/or for several physiological aspects (e.g. reflecting or adsorbing heat or other types of radiation; Caro 2005). When polychromatic individuals occur in different proportions in a population, negative frequency dependence may favour the maintenance of the rarer morphs, which provides stabilising selection for polymorphism (Galeotti et al. 2003; Roulin 2004).

Colour polymorphism is well-known in birds, which includes 33.3% of the species in the order Strigiformes and about 10% of Cuculiformes, Upupiformes, Galliformes, and Ciconiiformes (Galeotti et al. 2003). It is more frequent in lineages that live in both open and closed habitats and in species that show extended daily activity patterns, under variable light conditions (Galeotti et al. 2003). For example, the lighter, white morph of the barn owl (*Tyto alba*) is able to capture prey more efficiently under brighter moonlight conditions than the darker, reddish morph (San-Jose et al. 2019). Colour polymorphism may also be related to personality, as seen in the Gouldian finch (*Erythrura gouldiae*), where red-headed individuals were more aggressive than the black-headed morph, but black-headed individuals appeared to be bolder (i.e. approaching and touching novel objects more often) and less risk-averse (i.e. more likely to return to a feeder after the experimental presentation of a predator) (Williams et al. 2012).

**Fig. 1:**
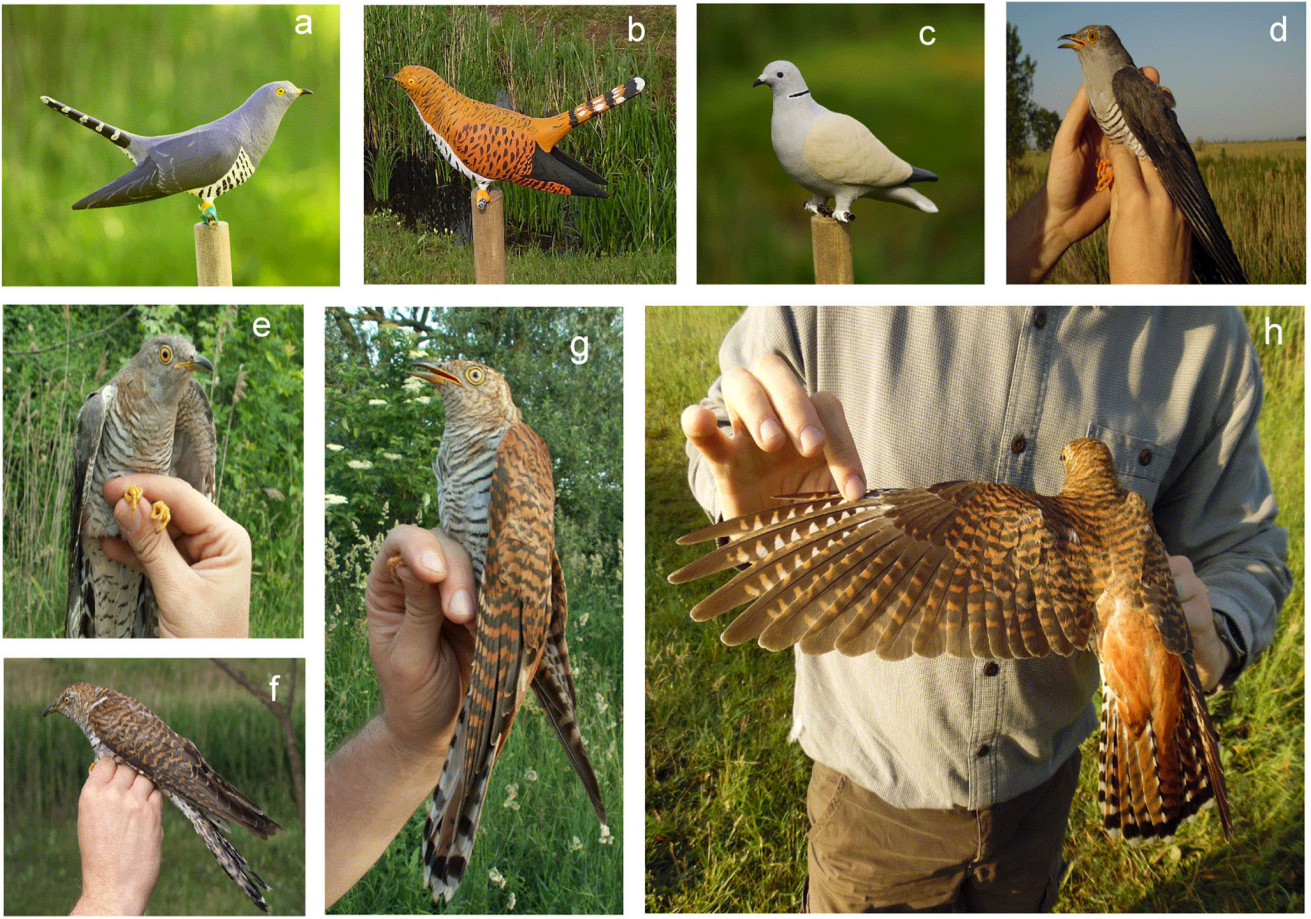
Colour variants of live common cuckoos (*Cuculus canorus*) and decoys used for model presentation experiments, including Eurasian collared dove (*Streptopelia decaocto*) used for a neutral control. Decoys: **a**: grey cuckoo, **b**: rufous cuckoo, **c**: collared dove; live common cuckoos: **d**: adult male, always grey; **e**: grey adult female, and a series of rufous adult females: **f**: brownish female; **g**: red-brown female; and **h**: bright orange-red female. Birds were caught by mist-netting at our study site in Hungary. Photo credits: (**a**) and (**c**): Zoltán Elek; (**b**), (**d**), (**f**) and (**h**): Csaba Moskát; (**e**) and (**g**): Miklós Bán

Colour polymorphism can be restricted to only one of the sexes in a species (Cuthill et al. 2017). However, this type of colour polymorphism is rare in female birds and occurs in only about 0.2% of avian species (Galeotti et al. 2003). Parasitic cuckoo species (*Cuculus* spp.) are one example of female-restricted, colour polymorphism in adults (Erritzøe et al. 2012; Payne 1967, 2005; Sato et al. 2015; Tanaka 2016). In these species, the ratio of eumelanin and pheomelanin plays a key role in generating grey and rusty colour morphs (Toral et al. 2008; see also in general McGraw et al. 2005; Ducrest et al. 2008). The common cuckoo (*Cuculus canorus*), a widely studied obligate brood parasite, is a typical example of adult female-specific, plumage polymorphism (e.g. Payne 2005; Mikulica et al. 2017; Wyllie 1981). This species meets the criteria defined by Galeotti et al. (2003) that predict the appearance of plumage polymorphism, i.e. it lives in a variety of habitats, which include semi-open habitats (Røskaft et al. 2002b), but shows a strong diurnal activity pattern (Moskát et al. 2019; Wyllie 1981; Yoo et al. 2019). Adult males are grey (monomorphic) and adult females are polymorphic independent of age, either individually grey (typically common in most cuckoo populations) or rufous (typically rare) (Fig. 1).

Most previous research focused on the potential role of cuckoo colour polymorphism in cuckoo-host relationships. Specifically, the grey morph of common cuckoos is thought to mimic the Eurasian sparrowhawk (*Accipiter nisus*), in what is known as the sparrowhawk mimicry hypothesis (e.g. Thorogood and Davies 2012; Gluckman and Mundy 2013). This sparrowhawk is a predator of small passerines, which includes cuckoo hosts, and this aggressive mimicry deters hosts from attacking the female cuckoo as a front-loaded antiparasitic defence strategy (Welbergen and Davies 2011). Interestingly, sparrowhawk mimicry cannot prevent cuckoos from being attacked by hosts altogether, neither in reed warblers (*Acrocephalus scirpaceus*) (Campobello and Sealy 2010; Welbergen and Davies 2011) nor in other passerine cuckoo hosts (Liang and Møller 2015; Moksnes et al. 1991; Røskaft et al. 2002a; Tryjanowski et al. 2018a, b), which include larger and more aggressive *Acrocephalus* species (Bártol et al. 2002; Dyrcz and Halupka 2006; Li et al. 2015; Ma et al. 2018; Marton et al. 2019). Although the rufous morph of female common cuckoos is somewhat similar to common kestrels (*Falco tinnunculus*) (the kestrel mimicry hypothesis, Voipio 1953), an experimental study rejected the idea that the rufous plumage of female common cuckoos was an adaptation to mimic this raptor species that also preys on small passerines (Trnka et al. 2015). At sites where the grey morph was common, the rufous morph received less aggression by hosts (Honza et al. 2006; Trnka and Grim 2013). However, this advantage disappeared when the rufous morph was more widespread in a population and had a similar frequency to that of the grey morph (Honza et al. 2006).

Apart from the well-studied role of colour polymorphism in cuckoo-host relationships, this polymorphism could also have intraspecific functions. First, adult female polychromatism may reduce sexual harassment of females by males in a reproductive and/or territorial context by preventing males from recognizing the rarer colour morph as a conspecific female individual. A recent study tested this hypothesis in an Asian population of common cuckoos where the rusty morph was nearly absent amongst adult females (Lee et al. 2019). In that study, which was conducted as a field experiment, males tried to copulate with female models of the grey colour morph more than with the rufous morph (Lee et al. 2019).

Female common cuckoos typically parasitise the nests of small songbirds within a specific area – an individual laying area - (Chance 1940; Wyllie 1981). However, individual laying areas of multiple females sometimes partly overlap (Moskát et al. 2019; Nakamura and Miyazawa 1997; Wyllie 1975). Female common cuckoos defend their laying area from rival conspecific females, which show at least some degree of territorial-like behaviour when guarding their potential host nests (e.g. Moskát and Hauber 2019). Researchers disagree on the degree of territoriality of female common cuckoos: this species has been defined by some researchers as strictly territorial (Dröscher 1988; Honza et al. 2002; Nakamura et al. 2005), but others termed it as mostly territorial (Gärtner 1981; Riddiford 1986) or non-territorial (Vogl et al. 2004). Nonetheless, a second hypothesis for the intraspecific role of female adult colour polymorphism suggested that this may help to reduce intrasexual aggression caused by the territorial-like behaviour of breeding females (Horton et al. 2012) that compete for critical resources: namely, available host nests within their laying areas. Although this second hypothesis has not yet been studied in common cuckoos, a study by Svensson et al. (2009) on lizards and damselflies concluded that female colour polymorphism functioned intraspecifically to avoid both intersexual harassment and intrasexual competition for critical resources.

Both sets of these hypotheses assume that the rarer morph, which is typically the rufous morph in common cuckoos, has an evolutionary advantage of not being mobbed/harassed as heavily as the more common grey female cuckoo morph (Lee et al. 2019; Li et al. 2015; Trnka and Grim 2013). For instance, the appearance of a novel predatory species may have caused an initial recognition failure in prey birds (Carlson et al. 2017; Vesely et al. 2016) and may have favoured the rarer morphs. Similarly, a recognition problem is expected to occur toward a new or rare colour variant of brood parasites by their hosts. The role of social learning by hosts to recognise parasites may further increase the adaptive benefit of the rarer colour morph in female cuckoos (Thorogood and Davies 2013).

Morph ratios of female cuckoos appear to vary geographically, and this variation has been thought to be related to ecological factors that keep them at equilibrium at these different ratios (Thorogood and Davies 2012). Overall, however, the typically rarer rufous colour variant does not appear to be growing in frequency in most cuckoo populations where it is tracked over time, even though it is thought to garner a greater fitness advantage than the more common grey morph (Mappes and Lindström 2012). Yet, in some populations, the rufous morph has become as common as the grey morph, including at our study site in Hungary (Honza et al. 2006). In such a stable but unbiased sex-ratio situation, it is assumed that each of the colour morphs has a specific fitness advantage over the other in regarding a specific physiological function (e.g. different costs of rusty vs. grey pigment production) and/or regarding an ecological context (e.g. camouflage from hosts in a specific microhabitat), and these effects are summed to yield similar fitness payoffs (Galeotti et al. 2003; Roulin 2004). Alternatively, when selection pressure is weak, different and even unsuitable alternative morphs may co-exist for extended periods due to stochastic effects, such as population perturbation, colonisation, or range expansion (Excoffier et al. 2009; Johanesson and Butlin 2017).

Although many aspects of brood parasitism are well understood in birds (Soler 2017), how brood parasitic birds recognise their conspecifics remains poorly understood (Göth and Hauber 2004). Only a handful of experiments have addressed the ontogeny and the mechanisms (e.g. the phenotypic and vocal cues) used by brood parasites to recognise their conspecifics (e.g. Soler and Soler 1999; Hauber et al. 2000; Payne et al. 2000; Louder et al. 2019). In this study, we specifically address a territorial conflict scenario that assumes that the rarer morph would have an advantage amongst adult female cuckoos when competing for critical breeding resources, such as host territories or nests. This advantage is expected to disappear when both colour morphs are widespread in a population, which is similar to the predator mimicry and sexual harassment hypotheses.

Here we tested this territorial conflict hypothesis (i.e. how female common cuckoos respond to the two female colour morphs by using playbacks and model (decoy) cuckoo presentations). We predicted strong responses to the cuckoo models relative to control (Eurasian collared dove *Streptopelia decaocto*) treatments, but we also predicted no preferential social responses toward either the grey or the rufous morphs because their frequencies were similar in our study population at Apaj, Hungary (Honza et al. 2006), and females would defend their resources (host nests) from as many females as possible, irrespective of morph colour. We also predicted that plumage colour discrimination is not biased by sex because males in our population should court and mate with as many females as possible, irrespective of colour. Regarding the dominant sensory modality of intraspecific communication, we hypothesised that acoustic cues play a primary role in both male and female cuckoos’ recognition of and responses to potential mates or intruders over visual cues and physical proximity. Thus, we predicted that male cuckoos would respond quickly to female cuckoo calls, first acoustically, and only then by approaching to inspect the newcomer visually. We also predicted that female cuckoos would try to avoid direct, physical aggressive contacts with intruding females and would respond primarily to unfamiliar female cuckoos’ bubbling calls acoustically. Given that in our study area the frequencies of the two colour morphs of female cuckoos were similar, our results could serve as reference for future similar studies with uneven frequencies of adult female cuckoo colour morphs.

## Study area and methods

The study was conducted in a 20 × 40 km area around the village of Apaj (47° 6′ 53.9″ N; 19° 5′ 21.2″ E), in central Hungary ca. 50 km south of Budapest. This area contained a dense network of narrow irrigation and flood-relief channels. The channels were typically surrounded by banks that were 2 to 5 m high and covered with trees and bushes on one or both sides of the channels. In this semi-open habitat cuckoos and their movements were easily visible to researchers. In this area, common cuckoos parasitised great reed warblers (*Acrocephalus arundinaceus*), which bred in 2- to 5-m-wide reed beds along both sides of the channels (Moskát and Honza 2000). The frequency of parasitism was high in the area (ca. 50% of nests had one or more cuckoo eggs; Zölei et al. 2015). Cuckoos parasitised this host species where trees hybrid poplars, white poplars (*Populus alba*), willows (*Salix alba*)*,* black locusts (*Robinia pseudoacacia*), and Russian olives (*Eleagnus angustifolia*) were present along the channels, which were used by cuckoos as perches to locate and to monitor breeding activities of potential hosts (Moskát and Honza 2000). Both sexes of adult cuckoos seem to have high intra- and interannual breeding site fidelity (Bán et al. 2018; Moskát et al. 2019). Rufous adult female plumage morphs were common in this cuckoo population, with a frequency of ca. 60% (Honza et al. 2006).

For playback experiments, we recorded female common cuckoo calls (“bubbling calls”) between 2015 and 2018. Bubbling calls are short (ca. 2 s long), sex-specific calls that are quite different from the “cu-coo” calls of males (Deng et al. 2019; Moskát and Hauber 2019; Xia et al. 2019). We also recorded calls of the Eurasian collared dove within our study area as control vocalisations during that same period. The collared dove is a harmless, sympatric species of cuckoos and their great reed warbler hosts, and they have often been used as controls for field experiments with common cuckoos as taxidermic mounts (e.g. Bártol et al. 2002; Davies and Welbergen 2008; Lovászi and Moskát 2004; Trnka et al. 2015) or as playback calls (Moskát et al. 2017; York and Davies 2017).

We coupled our playback experiment with the presentation of 3D plastic models that were printed on an Ultimaker 2+ 3D printer using standard white ColorFabb PLA filament. The source file of the life-size cuckoo model was supplied by 3D Quick Printing Service (Golden Green Barn, Sandpitts Lane, Coventry, UK). The source file of the dove was downloaded from Thingiverse (https://www.thingiverse.com/), which is a free repository for 3D models. Three common cuckoo models (two of the more variable rufous morph and one of the less variable grey morph) and two collared doves, acrylic-painted models were used for the experiments (Fig. 1) (see also Marton et al. 2019 for a description of the decoys). Although we only had one model specimen of the grey morph, which may have resulted in visual but not acoustic pseudoreplication, this same decoy had already been used in a previous experiment where great reed warbler hosts aggressively attacked it more than controls (Marton et al. 2019).

**Fig. 2:**
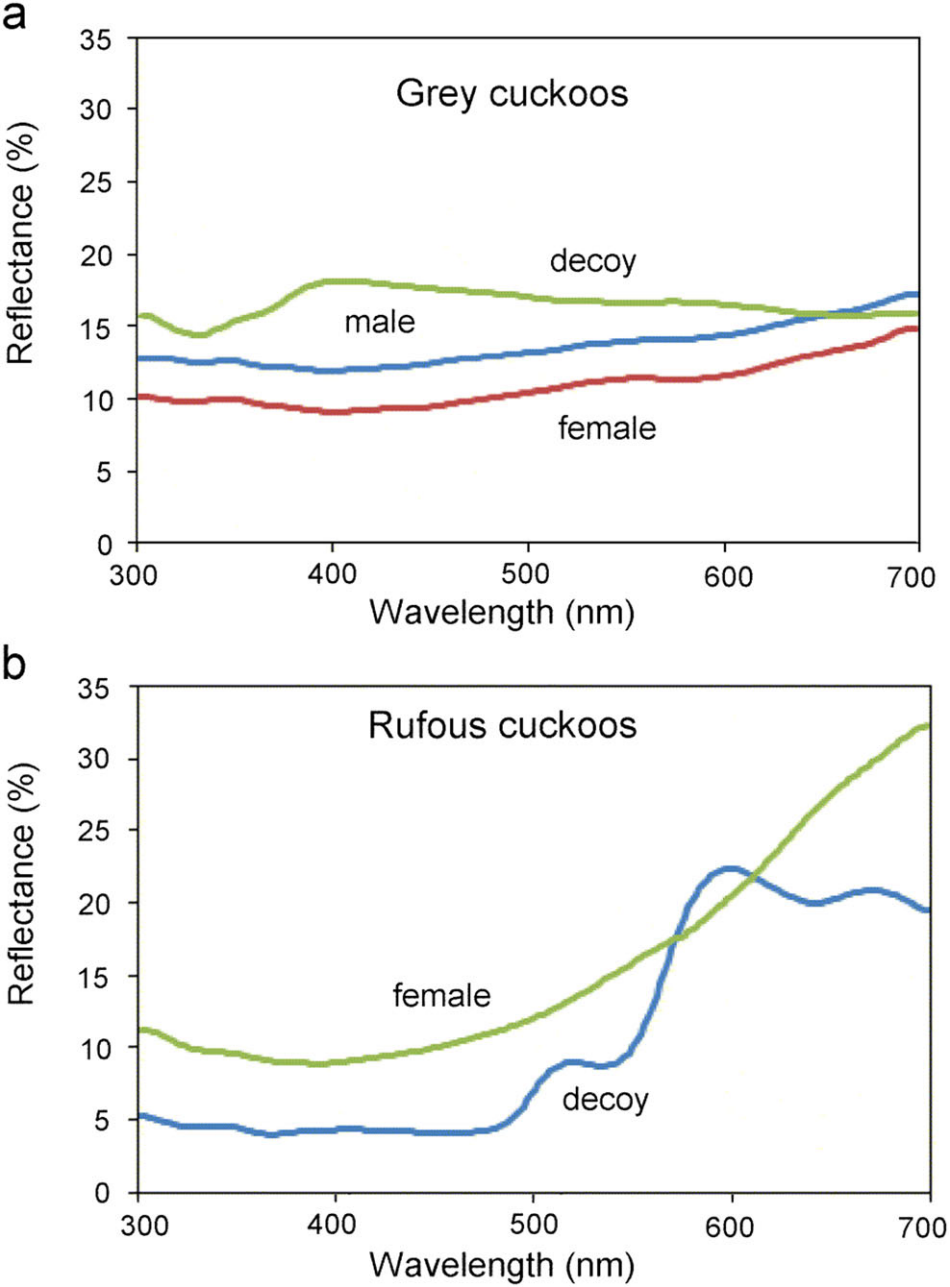
Spectral reflectance of common cuckoo (*Cuculus canorus*) feathers and 3D model cuckoos. (**a**): feathers of grey male and grey female, and grey decoy; (**b**): feather of rufous female and rufous decoy. All curves show mean values of six measurements. Reflectances are shown in the 300–700-nm interval as typical, although cuckoos are expected not to be sensitive for the ultraviolet range (i.e. < 380 nm; Aidala et al. 2012; for more details see “Study area and methods”)

The plumage colour of live rufous female cuckoos showed some variation, which ranged from brown to orange-reddish (Fig. 1). For our experiments, we chose the orange-reddish form because it was easily discernible by a researcher in the field. Many birds perceive a wider spectrum of light than humans (Stoddard and Hauber 2017) because they have a fourth, UV-sensitive cone in their retinas. However, the visually perceivable spectral range of cuckoos is likely more similar to humans, which was suggested by a genetic study of the short wavelength-sensitive type 1 (SWS1) opsin gene in shining cuckoos (*Chalcites lucidus*) and long-tailed cuckoos (*Urodynamis taitensis*) that indicated the presence of violet-sensitive (VS) and not ultraviolet-sensitive (UVS) cones (Aidala et al. 2012). The lack of UVS sensitivity was also supported indirectly through feather light-reflectance analyses that showed no strong reflectance in the UV range of the cuckoo’s plumage (Mullen and Pohland 2008; see also Koleček et al. 2019). Here, we measured avian-visible reflectances of adult cuckoos’ feathers and the 3D models (Fig. 2) with a USB 2000 spectrophotometer (Ocean Optics, Europe) with a DH-2000 deuterium light source and R400-7 bifurcated fibre-optic probe. The probe was oriented at a 90° to the surface (see for more details in Laczi et al. 2011).

The structure of our playback sound files was the same as the one used by our team in a recent similar study (Moskát and Hauber 2019). The short, 2-s female cuckoo bubbling call was repeated thrice in a 30-s period, followed by a 15-s break, and then repeated twice for a total duration of 2 min (without a final 15-s break). The last bubbling call unit was instead followed by a 2-min silent observational period (only the model bird was presented for visual cues). The same experimental design and playback file structure were used for the control stimuli of the dove models and calls.

We initiated a playback experiment within 2 min at a site after we heard the bubbling call of a female cuckoo ≤ 50 m away. We set up a loudspeaker (model: JBL Xtreme 40 W; volume was ca. 90 dB at 1 m distance measured by Voltcraft SL-100 sound meter by Conrad GmbH, Kalchreuth, Germany) on a tree at ca. 1–1.5-m height on the bank of the channel (typically above of the top of the reeds in the channel), which was connected by a 20-m audio cable to a Lenovo TAB 2 A7 tablet that contained the playback files in 16-bit .wav format.

We recorded our observations on cuckoos on a Tascam dr-05 ver2 sound recorder by verbally narrating the details of our observations. A second observer recorded cuckoo calling behaviours using a Marantz PMD-620 MKII audio recorder, a Sennheiser ME66 shotgun microphone, a FEL-MX mono preamp, an AKG K141 MKII headphone, a Rode PG2 pistol grip, and a Rode WS6 Deluxe windshield. After we adapted to the quick movements of the birds, distances of perching and flying cuckoos were estimated visually after observers had trained with a Bushnell Yardage Pro 800 rangefinder to estimate distances in the field.

Previous studies on common cuckoos that used VHF radio telemetry (Moskát et al. 2017) or GPS tags (Moskát et al. 2019) revealed that male common cuckoos maintained and defended territories from conspecific males during their breeding season in our study area. Similarly, female cuckoos also appeared to maintain territories during the breeding season, as evidenced by GPS data (Moskát et al. 2019) and playback experiments (Moskát and Hauber 2019). Here, we attempted to reduce the chance of collecting data on the same focal bird twice for the same type of trial. This is because “experienced birds” (Budka et al. 2019) may reduce their response or increase their response to repeated simulated territorial intrusions (Sprau et al. 2014). Therefore, we moved slowly by car from the first playback site along the irrigation channels to the next territory if we heard two females simultaneously calling from two such sites. In other cases, we moved by car > 1 km away along the channel, where we conducted the next experimental trial with a calling female if she was present. This pattern of site selection was implemented to reduce pseudoreplication (Hurlbert 1984; Kroodsma 1989). For the same reason, we used each playback file only once, and did not use multiple recordings from the same individuals.

We applied three different treatments: (i) rufous cuckoo model with female cuckoo bubbling calls, (ii) grey cuckoo model with bubbling calls, and (iii) collared dove model with dove calls. We observed the activity of cuckoos for 4 min (2-min playback and the next 2-min post-playback period) while hid behind bushes (see for more details in Moskát and Hauber 2019). For direct comparisons of responses to playbacks and model presentations, we used binary (yes/no) variables that expressed response/lack of response by wild cuckoos to the experimental trials whether they responded acoustically or by moving within the 50-m radius around the model bird. We also measured the following variables in the same way for both the experimental and the control trials: distance from loudspeaker at first detection (m), closest distance (m) during playback, time elapsed between the start of the trial and the time of the closest distance (s), the time of the first movement of the focal bird (“movement latency”, s), the time of the first calling by the focal bird (“calling latency”, s), the duration of continuous calling (s), the number of calls uttered, the number of flights toward the model, and the total number of cuckoos observed. We identified sexes by plumage (rufous: all females) and/or calls (bubbling calls: all females, cu-coo calls: all males). All variables were recorded for both sexes, except that the number of calls for males was replaced by the number of call types (see details in Moskát and Hauber 2019): these included the “cu-coo” advertising call (sensu Lei et al. 2005) and the mate attraction vocalizations of the quick “cu-cu-coo” (Lei et al. 2005; Xia et al. 2019) and “gowk” (Lei et al. 2005) calls, respectively (latter category also included the difficult-to-distinguish “guo” calls (sensu Wyllie 1981)).

For simple bivariate comparisons of trials (reaction compared with no reaction), we applied categorical tests (Fisher’s exact and χ^2^ tests). In turn, we used logistic, generalised linear models to study the relationship between behavioural variables that were considered fixed factors and occurrences of focal bird displays as the response variable. We applied the “binomial” family of distribution for occurrence data using the complementary loglog link function. This link function is asymmetric and will often produce different results from the logit and probit link functions. The complementary loglog corresponds to applications where we can detect either zero events (e.g. defects) or one or more event, where the number of events is assumed to follow the Poisson distribution (Van Horn 2015). We modelled these data with generalised linear models (GLM, Bolker et al. 2009) using the glm function in R 3.6.1 (R Core Team 2019) and the following explanatory variables in the evaluated models: (i) time-based variables (measured in seconds): time at first detection, time at closest detection, latency of calling, length of continuous calling; (ii) distance-based variables (measured in metres): distance at first detection, closest distance; and (iii) meristic variables: number of calls, number of flights, number of birds. For the parameterisation of the most parsimonious model, we used a model selection information criterion (AICc) to rank the above models in terms of their ability to explain occurrences while accounting for the number of parameters estimated (Burnham and Anderson 2002). First, we fitted a full logistic model that included all explanatory variables mentioned above, and then we removed the variable with the least explanatory power, refitted the model, and repeated this process until we reached the optimal number of model parameters based on AICc (Bolker et al. 2009). In this way, a “best approximating” model was selected as the most parsimonious explanation of the data. We conducted this model parameterisation approach for each sex separately.

Behavioural and acoustic responses of female and male cuckoos were analysed separately using principal component analysis (PCA) in the program package SPSS ver. 17 (SPSS Inc., Chicago, IL, USA). PCAs were run on the correlation matrix of response variables, and components were retained where the corresponding eigenvalues were > 1.0. No subsequent rotation on component loadings was applied.

## Results

### Responses of cuckoos to rufous and grey cuckoo models coupled with bubbling call playbacks

The simple categorisation (see “Study area and methods”) of the output of our trials revealed that both female and male common cuckoos showed consistently more responses toward the cuckoo playbacks and models than the control doves (Table 1). Cuckoos frequently responded to conspecific models and playbacks by approaching movements and calling behaviour. Female cuckoos responded to the rufous morph model and playbacks in 14/18 of trials, and male cuckoos responded in 16/18 cases (both sexes responded in 12/18 cases). Similar response frequencies were obtained when using the grey cuckoo model coupled with the playback: 15/17 responses by females and 15/17 responses by males, with 13/17 trials when both sexes responded.

**Table 1:** Summary of female and male responses of common cuckoos (*Cuculus canorus*) to vocal playbacks of female cuckoo calls coupled with presentations of different models (rufous female cuckoo, grey female cuckoo, and Eurasian collared dove (*Streptopelia decaocto*) used for control)

	Acoustic response or movement	Acoustic response	Flights	N
Female cuckoo responses to				
Rufous cuckoo model	14	13	13	18
Grey cuckoo model	15	13	13	17
Dove control model	4	4	4	17
Male cuckoo responses to				
Rufous cuckoo model	16	15	16	18
Grey cuckoo model	15	14	14	17
Dove control model	5	4	4	17

**Fig. 3:**
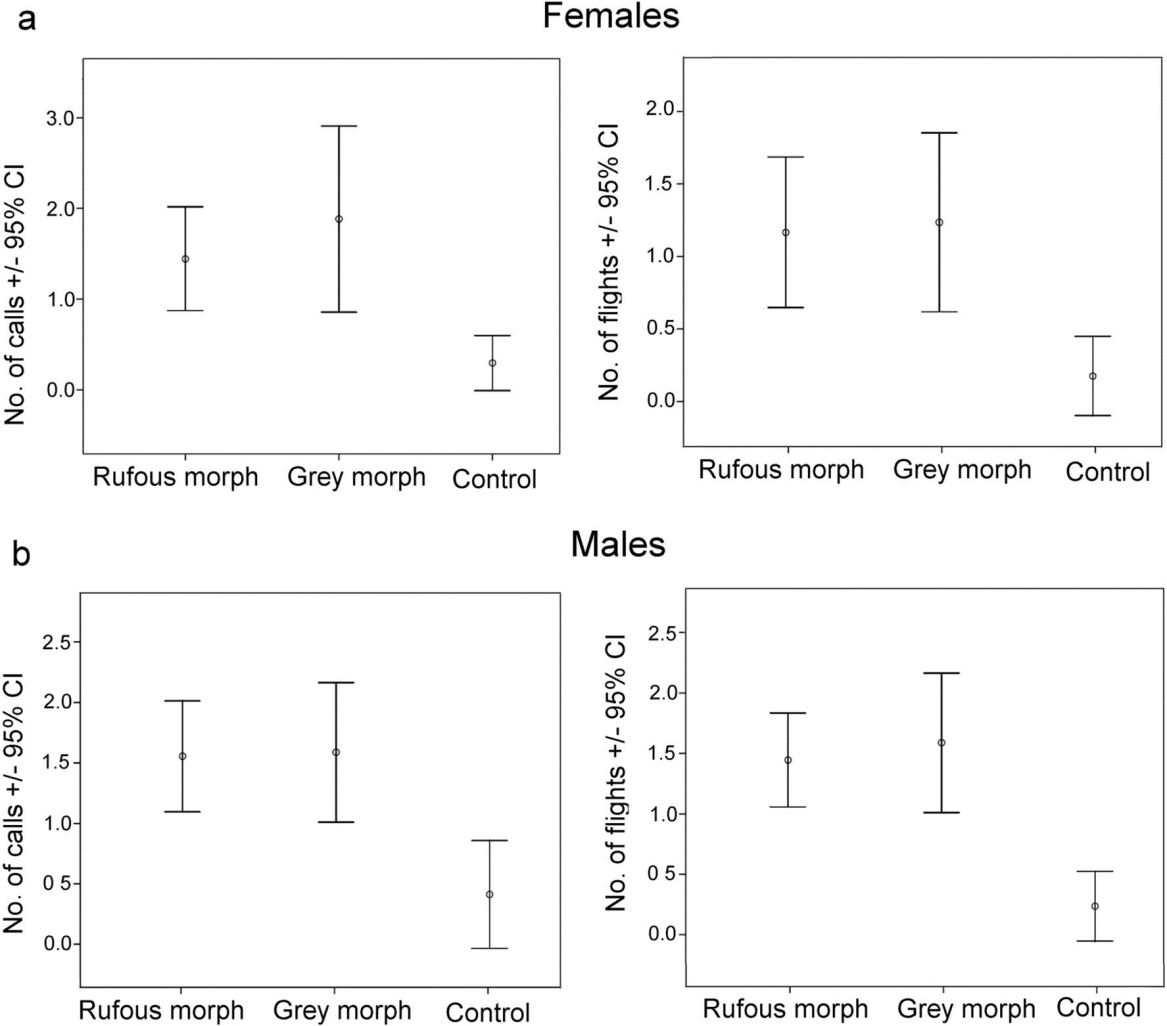
Acoustic (number of calls) and movement (number of flights) responses of female (**a**) and male (**b**) common cuckoos (*Cuculus canorus*) to playback experimental trials where female cuckoo bubbling calls were played back with the demonstration of a rufous or grey model of cuckoos, relative to controls (Eurasian collared dove (*Streptopelia decaocto*) calls with a collared dove model). Means and 95% confidence intervals are shown

Critically, the variation in wild cuckoo responses to the playbacks of female cuckoo calls coupled with either colour morph of the cuckoo model and the control dove presentations was significant for both cuckoo sexes (Fisher’s exact tests, both *P* < 0.01), but the responses to the two types of colour morph models were statistically similar (rufous vs. grey: *P* = 0.658 for females, and *P* = 0.677 for males). Similar patterns were found when acoustic responses were analysed separately from movement responses, and also when the numbers of flighted approaches toward the model were compared solely (Fig. 3). Both female and male cuckoos also responded vocally more intensively to cuckoo presentations than to the dove controls (all *P* < 0.001). In turn, cuckoos responded vocally and with number of flights equally to the two colour morphs (females: *P* = 1.0, males: *P* = 1.0).

### Behaviour of female and male cuckoos during the experiment

**Fig. 4:**
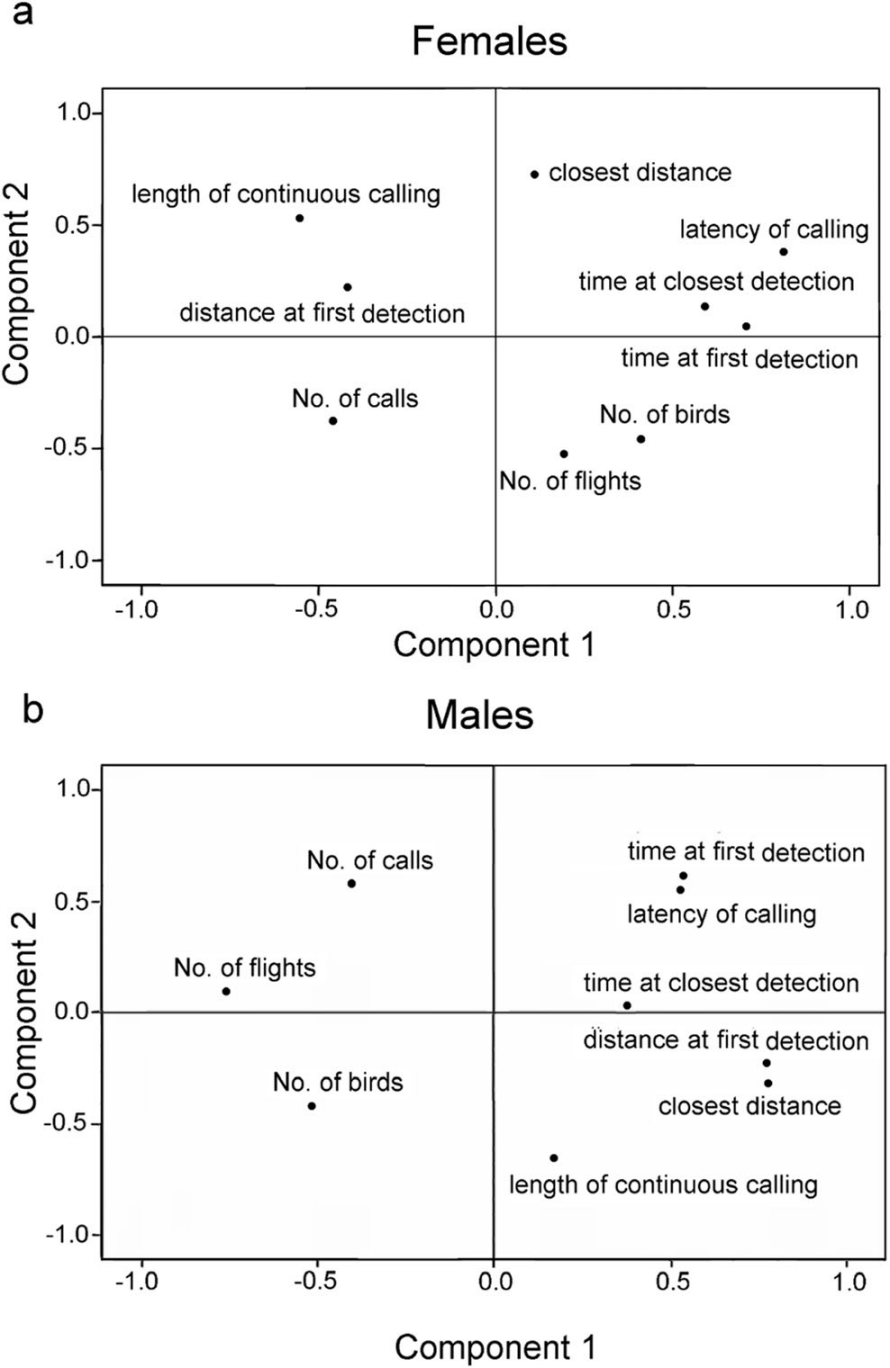
PCA ordination plots of response variables of female (**a**) and male (**b**) common cuckoos (*Cuculus canorus*) to playbacks of female cuckoo bubbling calls and with grey and rufous colour morphs of model cuckoos. The component loadings are shown for the first two principal components

**Fig. 5:**
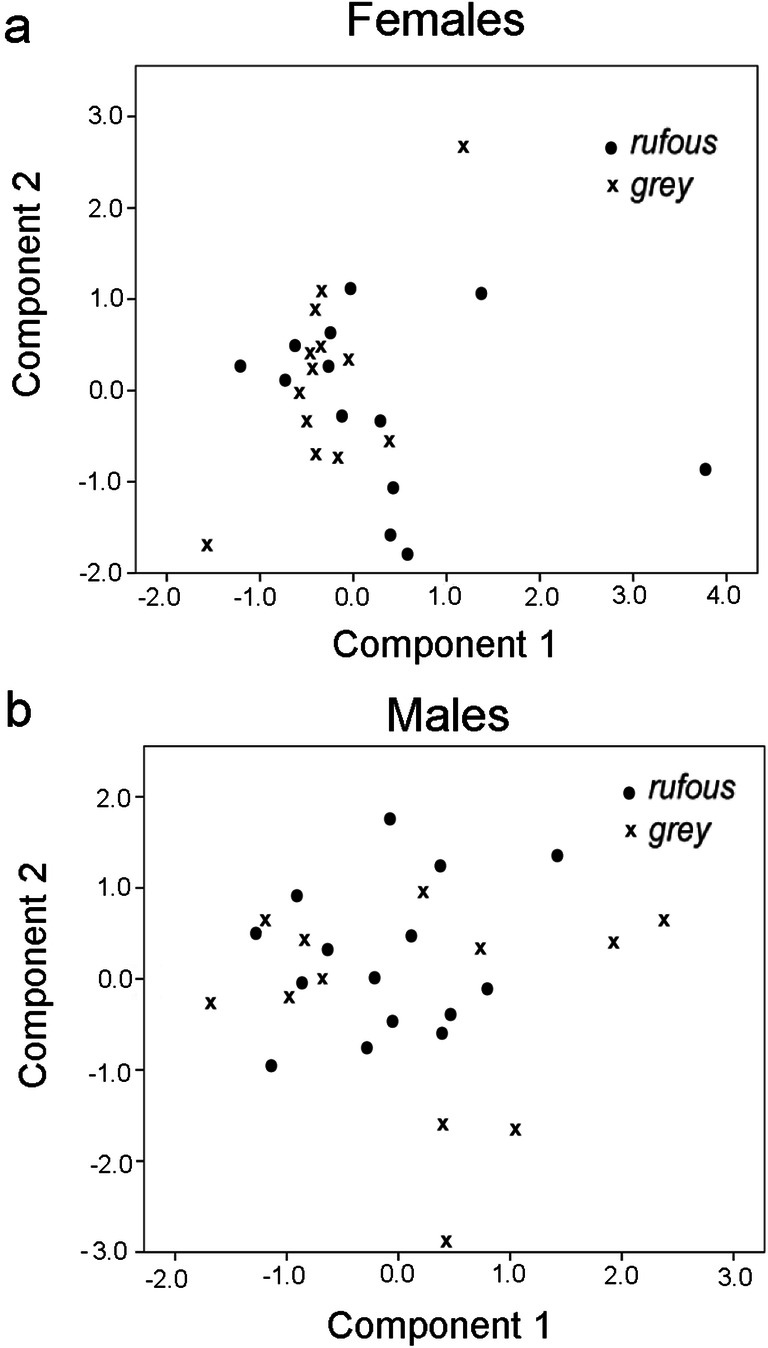
PCA score plots of responses of female (**a**) and male (**b**) common cuckoos (*Cuculus canorus*) to playbacks of female cuckoo bubbling calls and with grey and rufous colour morphs of model cuckoos

Neither logistic regression models (Table 2) nor the PCAs (Table 3; Figs. 4a, b and 5a, b) revealed statistical differences in how female and male cuckoos responded to grey compared with rufous cuckoo models. Stepwise logistic regression retained the variables time of closest detection, latency of calling, maximal continuous calling, and the number of flights by females, and it retained distance in first detection, closest distance, and time of closest detection by males (Table 2). The PCAs also revealed sex-specific characteristics of cuckoo behaviour in our experiment. For example, in females, the variables of time at first detection and latency of calling showed the highest positive loadings for component 1, whereas distance at first detection and closest distance in males with positive signs and the number of flights with negative sign in males (Table 3).

**Table 2:** Responses of female and male common cuckoos (*Cuculus canorus*) to vocal playbacks of female cuckoos coupled with different colour morph models, based on a logistic generalized linear regression. The unit of measurement of each variable is indicated in parentheses

Focal birds’ sex	Variable	Estimate	S.E.	*z*	*p*
Female	Intercept	0.039	1.005	0.039	0.969
	Time at closest detection (s)	− 0.002	0.004	− 0.535	0.592
	Tatency of calling (s)	0.004	0.005	0.831	0.406
	Maximum length of continuous calling (s)	− 0.465	0.335	− 1.387	0.166
	No. of flights	0.308	0.304	1.015	0.310
Male	Intercept	0.025	0.533	0.048	0.962
	Distance at first detection (m)	− 0.020	0.033	− 0.590	0.552
	Closest distance (m)	0.002	0.037	0.060	0.952
	Time at first detection (s)	0.0004	0.004	0.100	0.915

**Table 3:** Component matrix of PCAs on female and male common cuckoos’ (*Cuculus canorus*) responses to the cuckoo model presentations with playbacks of female cuckoo bubbling calls. The unit of measurement of each variable is indicated in parentheses

	Component						
	Females			Males			
	PC1	PC2	PC3	PC1	PC2	PC3	PC4
Distance at first detection (m)	− 0.424	0.221	0.762	0.773	− 0.228	0.185	0.438
Time at first detection (s)	0.858	0.042	0.090	0.534	0.609	0.107	0.071
Closest distance (m)	0.116	0.724	− 0.179	0.777	− 0.319	− 0.158	0.358
Time at closest detection (s)	0.590	0.137	0.569	0.376	0.024	0.873	0.043
No. of calls	− 0.461	− 0.377	0.111	− 0.403	0.577	0.337	0.427
Latency of calling (s)	0.820	0.372	− 0.024	0.530	0.551	− 0.013	− 0.232
Lengths of continuous calling (s)	− 0.563	0.530	0.361	0.175	− 0.656	0.530	− 0.410
No. of flights	0.193	− 0.525	0.104	− 0.758	0.094	0.500	0.046
No. of birds	0.410	− 0.457	0.450	− 0.513	− 0.422	− 0.044	0.526
Eigenvalue	2.684	1.639	1.302	2.939	1.779	1.480	1.009
Cumulative variance explained (%)	29.82	48.03	62.50	32.66	52.42	68.87	80.08

## Discussion

In our study area in Hungary, we did not find any behavioural or acoustic selectivity in adult cuckoo responses between the presentations of either of the two colour morphs of conspecific models coupled with playbacks of female bubbling calls. Both males and females responded to the different morphs similarly. In an experiment performed in South Korea, male common cuckoos also treated the grey and rufous females similarly, except that they tried to copulate more frequently with the grey colour variant (Lee et al. 2019). However, the rufous morph was extremely rare in Korea and was practically lacking from many areas (e.g. it was represented by none of 18 females studied by Noh et al. 2016), and so, at that site, it probably represented an entirely novel colour variant when tested with male common cuckoos. Although male cuckoos in our study did not attempt to copulate with female models, the similar intensity of responses to both colour morphs of female adults was consistent with Lee et al.’s (2019) prediction based on their sexual harassment hypothesis for a population without any “rare” morphs present.

In general, plumage colour of birds is influenced by environmental and intrinsic factors; for example, plumage colour may change with advancing age, seasonally, due to parasite infection, and/or with variation in body condition (e.g. Badás et al. 2018; Delhey et al. 2006). In contrast, colour polymorphs are typically genetically encoded in birds, but see age-dependence in female tree swallow (*Tachycineta bicolor*) colouration (Hussell 1983) and delayed plumage maturation in many male birds (Hawkins et al. 2012). Frequencies of colour polymorphisms in a population seem to be relatively stable, and colour polymorphism often has adaptive value for reproduction for the rarer morph, its behaviour, and/or life history (Roulin 2004). For example, in the tawny owl (*Strix aluco*), female colour plumage polymorphism was associated with their reproductive strategy in that grey females produced offspring of higher quality than rufous females but they did not breed every year (Roulin et al. 2003). In contrast, in the present study, we revealed that the rufous morph of female cuckoos seemed to have no recognition advantage over the grey morph, at least from the viewpoint of territorial intrusions by conspecifics of either sex. However, we cannot exclude its potential role in other aspects of cuckoo breeding behaviour, which include host-evasion.

We showed that female common cuckoos pay close attention to intruding females in their territories and responded both acoustically with bubbling calls and visually by approaching the decoys. Previously there was only limited information on inter-female aggression in common cuckoos, which included scarce observations on direct female-female fights (Moskát and Hauber 2019). Riddiford (1986) observed that territory-holder female cuckoos expelled intruding non-territorial females. More recently, however, Lee et al. (2019) reported four cases when 3D female common cuckoo dummies were attacked by adult female cuckoos in a field experiment. After observing cuckoos equipped with radio transmitters, Dröscher (1988) stated that female cuckoos defended their laying territories from other females, especially in the morning. In our study population, we observed an arriving female cuckoo chased another female that was already perched on a tree. In another case, a flying female cuckoo was attacked in mid-air when it was in the proximity of another female cuckoo (C. Moskát pers. obs.). Probably there are many more such observations from different cuckoo populations, but there is a problem of identifying the sex of adult grey cuckoos in the field accurately, except when these have been caught and marked with visible marks a priori, sexed morphologically or by DNA, and/or when radio telemetry or other tagging had been applied. Alternatively, whereas the sex of the rufous morph is clear (always female), the sex of the grey morph can still be identified when it produces male- or female-specific call types.

Our results demonstrated the importance of acoustic signals in inter-female recognition. In a recent study on the same population, we showed that female cuckoos responded to playback of females’ bubbling calls (Moskát and Hauber 2019). In that experiment, about half of the female cuckoos moved closer to the speaker, which suggested that they were directing attention toward the simulated intruder. In the current study, the intensity or frequency of responses did not increase when a model cuckoo was placed at the speaker. In contrast to Lee et al. (2019), we did not observe any direct contact (e.g. mobbing or copulation attempt) with the cuckoo model, but the goals and the experimental protocols differed between the two studies. Lee et al. (2019) placed dummy cuckoos for a longer period at a site and left them for 20 min after the first positive response was observed. We studied female cuckoos’ interest toward the appearance of new, unfamiliar conspecific females by following the protocol used in our previous study on female-female acoustic communications (Moskát and Hauber 2019). Our 4-min observational period was suitable to detect an acoustic response from male and female cuckoos and to attract their interest in the dummy cuckoo, which differed from the reactions toward the dove dummy used for control. Additionally, the type of dummy may result in a different intensity of responses toward the dummy. For example, Němec et al. (2015) revealed that red-backed shrikes (*Lanius collurio*) mobbed the dummy of the nest predator Eurasian jay (*Garrulus glandarius*) at various frequencies at their nests, which depended on the texture of the surface of the decoy presented. They behaved most aggressively toward a taxidermic mount, but the frequency of the attacks decreased toward a plush decoy, and the silicon decoy was attacked only when it was presented after the stuffed mount or the plush dummy.

Common cuckoos exhibit highly developed social lives (Davies 2000), although several details have not yet been described and understood. This species is thought to be polygynandrous (Marchetti et al. 1998; Wyllie 1975), where, in the simplest case, overlapping territories of 1–3 males encompass the laying area of a single female cuckoo. In our study area, we also found a similar spacing pattern of common cuckoos of overlapping territories of 1–3 males around an individual female cuckoo during their breeding season (Moskát and Hauber 2019). Male cuckoos that compete for females somewhat tolerate the presence of 1–2 additional (and presumably familiar) males in a female’s laying area, but are intolerant toward non-familiar intruders (Moskát et al. 2017).

We agree with Gärtner (1981) that the most typical case of a female territorial system is where dominant females have more or less separate (partly overlapping) laying areas. In such a system, it would be crucial that female cuckoos recognise each other, advertise their claim for territories, and defend their territories. Because common cuckoos exhibit no parental care and defending an area with several host nests suitable for parasitism is costly, behavioural mechanisms to lower the intensity and costs of inter-female aggression are predicted to evolve. Female calling could be seen as such a mechanism because it elicits a quick response from male and female cuckoos locally. The difference revealed by PCAs for females and males was explained by the higher number of males than females at a trial site and by the more cryptic behaviour of the females in this species (Davies 2000). In essence, these characters explained the quicker responses of males than females to decoys coupled with the playback calls. Besides the latency and intensity of responses, the presumed behavioural functions of the two sexes when detecting and intercepting an intruder during the experimental trial were also different. Males were likely attempting to look for new mating possibilities, whereas females were trying to defend their existing resources (i.e. host nests).

Theory predicts that the typically rare cuckoo female colour morph (i.e. rufous) can be evolutionarily advantageous from several aspects (e.g. Mappes and Lindström 2012). However, we report statistically similar responses of female and male cuckoos toward rufous and grey models of cuckoos presented during playback experiments with female bubbling calls when the rufous morph was as common as the grey morph. Our study revealed that colour polymorphism did not affect territorial interactions amongst female cuckoos and social interactions with males. Therefore, we suggest that further studies should address the role of colour polymorphism in other socio-ecological contexts. This could include sexual selection, immunity trade-offs, and parasite loads (Ducrest et al. 2008; Arai et al. 2018). In brood parasite-host interactions, adaptations and counteradaptations from the two sides are of particular importance. Future studies should also focus on cuckoo-host interactions in the context of adult female colour polymorphism both at sites where the rufous morph is rare (most populations) and again in Hungary (where the rufous morph is common). For example, female cuckoos often parasitise host nests in the late afternoon (e.g. Davies and Brooke 1988; Honza et al. 2002), or even under dim light conditions at sunset (our observations in our study area), when the rufous morph could be less visible to hosts and, consequently, could be more advantageous for laying rufous cuckoos.

## Data Availability

The datasets generated during and/or analysed during the current study are available from the corresponding author upon reasonable request.
